# Postpartum Stanford Type A Aortic Dissection: A Case Report and Review of the Literature

**DOI:** 10.4021/cr276w

**Published:** 2013-07-11

**Authors:** Esra Balbay, Semih Basci, Irem Bozkurt, Abdullah Ozkok, Sinem Dogruyol, Emel Erkus Sirkeci, Aytekin Oguz

**Affiliations:** a Department of Internal Medicine, Istanbul Medeniyet University, Goztepe Training and Research Hospital, Istanbul, Turkey; b Department of Emergency Medicine, Istanbul Medeniyet University, Goztepe Training and Research Hospital, Istanbul, Turkey

**Keywords:** Aortic dissection, Pregnancy, Postpartum

## Abstract

Acute aortic dissection is a life-threatening disease. Approximately half of the aortic dissection observed in women under 45 years old has been reported to be related to pregnancy. Herein, we present a case of type A aortic dissection diagnosed in postpartum period. A 37-year-old woman admitted to the emergency department with the complaint of sudden onset of dyspnea. Arterial blood pressure was measured as 170/100 mmHg in left arm and 90/60 mmHg in right arm. With a prediagnosis of aortic dissection, thoracic and abdominal computed tomograpy was performed and type A aortic dissection extending form carotid artery to renal arterial level was detected. Operation of aortic dissection together with coronary arterial repairment and aortic valve replacement were successfully performed. Aortic dissection is not uncommon in pregnancy and furthermore it is potentially life-threatening for both mother and fetus. A high level of suspicion is required for prompt diagnosis and treatment in the peripartum period.

## Introduction

Acute aortic dissection is a life-threatening disease with an incidence of 2.6 - 3.5 per 100.000 person-years [1, 2]. Well-known risk factors predisposing to aortic dissection include hypertension [3, 4], atherosclerosis, preexisting aneurysm, vasculitis, previous aortic valve replacement [5], collagen synthesis disorders such as Marfan and Ehler-Danlos syndromes [6], bicuspid aortic valve [7], aortic coarctation, cardiac catheterization [8] and trauma [9].

Approximately half of the aortic dissection observed in women under 45 years old has been reported to be related to pregnancy [10]. Although the exact pathophysiological mechanisms underlying the relationship between pregnancy and aortic dissection are still unknown, it has been hypothesized that hormonal and physiologic changes of pregnancy may induce and accelerate the development of aortic dissection [10, 11].

Herein, we present a case of Stanford type A aortic dissection diagnosed in postpartum period successfully treated together with coronary artery repairment and aortic valve replacement and also review of the literature concerning the aortic dissections observed in the peripartum period.

## Case Report

**Figure 1:**
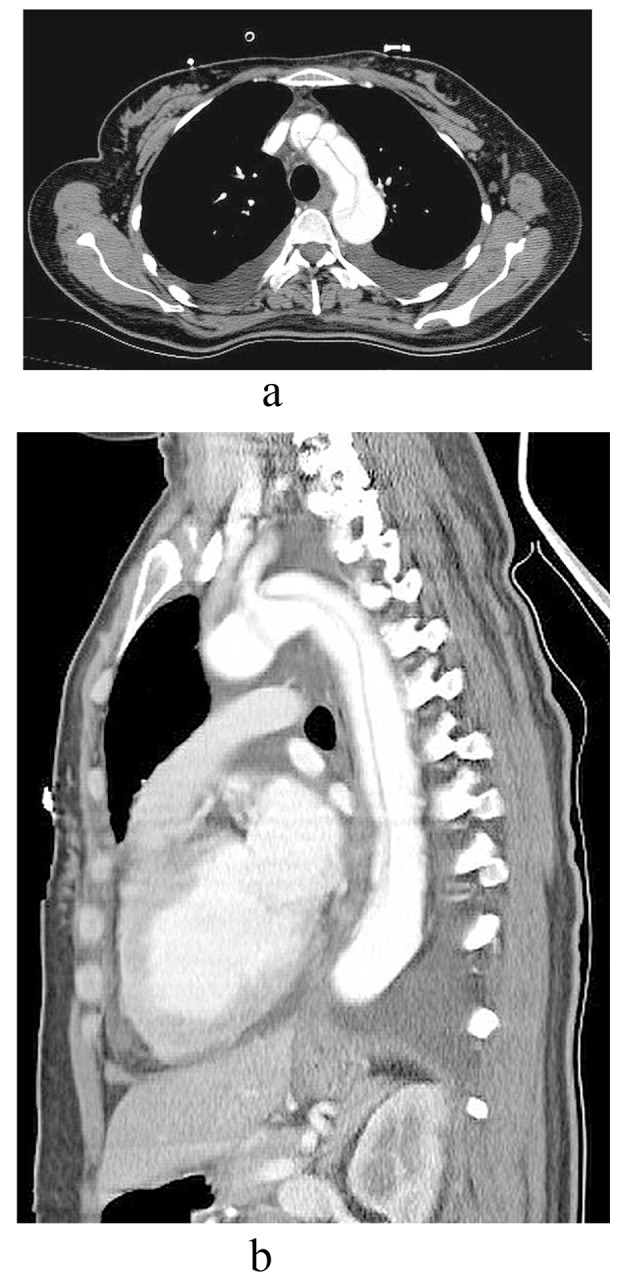
Stanford type A aortic dissection involving the proximal aorta with a prominent intimal flap; a: transverse plane, b: saggital plane.

A 37-year-old woman admitted to the emergency department with the complaint of sudden onset of dyspnea. She did not have any chest or back pain. She had no known chronic disease or recent drug use. She gave birth to a healthy baby with cesarean section two days ago. No complication occurred in the postoperative period. Pregnancy was also uneventful with normal blood pressure and glucose tolerance. In clinical examination, the patient was tachycardic and tachypneic. Arterial blood pressure was measured as 170/100 mmHg in left arm and 90/60 mmHg in right arm. Pulses were relatively weak in the right arm compared to left arm. Diffuse fine rales were present on the auscultation of lungs. No heart murmur was heard. She had bilateral mild pretibial edema. Electrocardiography revealed sinus tachycardia with incomplete right bundle branch block. In arterial blood gas analysis, respiratory alkalosis with hypoxia and hypocarbia (pH: 7.50, saO2 70%, pCO2: 30) was found. She was promptly monitorized and 3 liters/min of oxygen was started. Treatment with diuretics (furosemid 100 mg iv bolus) and nitroglycerine (Glyceryl trinitrate 40 µg/h intravenous infusion) were also given for presumed pulmonary edema. With a preliminary diagnosis of aortic dissection, thoracic and abdominal computed tomograpy (CT) with IV contrast agent was performed. Stanford type A aortic dissection extending form common carotid artery to renal arterial level was detected (Fig.1a, b). The patient was immediately transferred to the intensive care unit of department of cardiothoracic surgery. Operation of aortic dissection together with coronary arterial repairment and aortic valve replacement were successfully performed. Currently, patient is followed up by our cardiology and cardiovascular surgery outpatient clinics without complaints.

## Discussion

Acute aortic dissection is a life-threatening disease with a very high rate of cardiovascular morbidity and mortality. The most important and common risk factor is the systemic hypertension which has been reported in the 70% of the patients with aortic dissection [12]. In histologic studies, loss of corrugation of the elastic fibers, fragmentation of the reticulin fibers and decrease in the amount of acid mucopolysaccharides have been determined in patients with aortic dissection [11].

Most of the aortic dissection observed in young women has been reported to be related to pregnancy [10]. Pregnancy is considered to be an independent risk factor for aortic dissection however underlying mechanisms are not completely known. Possible mechanisms may be classified as mechanical and hormonal factors. Mechanical factors observed in pregnancy include the increase in heart rate, cardiac output, stroke volume, left ventricular mass and end diastolic volume [12]. In the third trimester, hemodynamic stress is maximal. Also with increasing gestational age, the compression on aorta and iliac vessels progresses because of the gravid uterus [13]. Hormonal factors in the course of pregnancy include the increased serum estrogen and progesterone levels which cause dilatation of great arteries including aorta. Receptors of estrogen and progesterone have been observed in aorta [14]. The presence of aortic root enlargement (> 4 cm) or increasing size of the aortic root during pregnancy have been reported as a risk factor for aortic dissection [15, 16].

We have reviewed the literature in terms of aortic dissections observed in peripartum period (Table 1). Accordingly, most of the dissections are type A (76%). The risk of aortic dissection seems to increase markedly in the later stages of the pregnancy especially in the 3rd trimester and also in the postpartum period in which hemodynamic stress is the most significant.

**Table 1:** Review of the Literature in Terms of Aortic Dissection Observed in Peripartum Period

	Age	Partum	Dissection Type
Survived	20 - 29: 12 30 - 39: 63 > 40: 3	1. Trimester: 1 2. Trimester: 3 3. Trimester: 55 Postpartum: 18	Type A: 57 Type B: 21
Maternal death	20 - 29: 1 30 - 39: 8 > 40: 1	1.Trimester: - 2.Trimester: - 3.Trimester: 8 Postpartum: 2	Type A: 10 Type B: -

Since mortality increases dramatically every hour when the diagnosis and treatment of aortic dissection are not performed, it is very important to make differential diagnosis quickly in such cases. Acute coronary syndrome, aortic aneurysm, pulmonary embolism, musculoskeletal pain, pericarditis and pleuritis should be included in the differential diagnosis of aortic dissection.

Selection of treatment modality in aortic dissection is based on the type of dissection. In Type A dissection if gestation age is before 30 week, aortic repair in utero is recommended. After 30 weeks of gestational age, cesarean section followed by surgery is the preferred treatment modality. Type B dissection is usually asymptomatic rather than Type A dissections and probably many patients are overlooked. In Type B dissection, medical treatment is the first choice and involves nitrates and β-blockers combination. Pregnant patients with high-risk of aortic dissection are recommended to be hospitalized between 28 and 32 weeks of gestational age [17-20].

In conclusion, aortic dissection is not uncommon in pregnancy and furthermore it is potentially life-threatening for both mother and fetus. A high level of suspicion is required for prompt diagnosis and treatment in the peripartum period.
